# The role of emotion dysregulation in the relationship between narcissism and suicide

**DOI:** 10.1192/j.eurpsy.2021.1562

**Published:** 2021-08-13

**Authors:** S. Beomonte Zobel, A. Sciarretta, P. Velotti

**Affiliations:** 1 Dynamical And Clinical Psychology, Sapienza Università di Roma, Rome, Italy; 2 Mental Health, Asl Roma 5, Tivoli, Italy

**Keywords:** emotion dysregulation, Suicide ideation, narcissistic vulnerability, Narcissism

## Abstract

**Introduction:**

Suicide attempts and suicidal ideation are peculiar aspects of several cluster b disorders, including Narcissistic Personality Disorder. Similarly, difficulty in regulating negative affects can play a role in the relationship between narcissist features and suicidal ideation. To date, it is still unclear which facet of narcissism is more related to the desire to die and which other factors are involved in this relationship.

**Objectives:**

To offer preliminary empirical evidences concerning the relationship between narcissism, emotion regulation and suicide ideation.

**Methods:**

We administered Pathological Narcissism Inventory (PNI), Difficulties in Emotion Regulation Scale (DERS) and Beck Scale for Suicidal Ideation (BSI) to a sample of individuals with Suicide ideation (n= 68) and a sample of community participants (n=140).

**Results:**

Controlling for age and gender, we found that BSI scores correlated significantly with the vulnerable dimension of narcissism, but not with the grandiose one, and with all DERS dimensions. Nevertheless, emotion dysregulation moderates the relationship between vulnerable narcissism and suicidal ideation.

**Conclusions:**

Suicide ideation seems to be deeply connected with the vulnerable dimension of pathological narcissism and the relationship between the constructs is partially mediated by emotion dysregulation. Future directions and clinical implications are discussed.

